# Micro-costing analysis of suspected lower respiratory tract infection care in a French emergency department

**DOI:** 10.3389/fpubh.2023.1276373

**Published:** 2023-10-04

**Authors:** Valérie Wilmé, Érik-André Sauleau, Pierrick Le Borgne, Éric Bayle, Pascal Bilbault, Sabrina Kepka

**Affiliations:** ^1^Emergency Department, Strasbourg University Hospital, Strasbourg, France; ^2^Public Health Department, Strasbourg University Hospital, Strasbourg, France; ^3^ICube Laboratory, French National Center for Scientific Research (CNRS), UMR 7357, University of Strasbourg, Illkirch-Graffenstaden, France; ^4^Regenerative NanoMedicine (RNM) and Federation of Translational Medicine (FMTS), French National Institute of Health and Medical Research (INSERM), UMR 1260, University of Strasbourg, Strasbourg, France

**Keywords:** cost analysis, emergency department, respiratory infections, health economics, micro-costing

## Abstract

**Introduction:**

In the context of budgetary constraints faced by healthcare systems, the medical-economic evaluation of care strategies becomes essential. In particular, valuing consumed resources in the overcrowded emergency departments (EDs) has become a priority to adopt more efficient approaches in treating the growing number of patients. However, precisely measuring the cost of care is challenging. While bottom-up micro-costing is considered the gold standard, its practical application remains limited.

**Objective:**

The objective was to accurately estimate the ED care cost for patients consulting in a French ED for suspected lower respiratory tract infection.

**Methods:**

The authors conducted a cost analysis using a bottom-up micro-costing method. Patients were prospectively included between January 1, and March 31, 2023. The primary endpoint was the mean cost of ED care. Resources consumed were collected using direct observation method and cost data were obtained from information available at Strasbourg University Hospital.

**Results:**

The mean cost of ED care was €411.68 (SD = 174.49). The cost elements that made the greatest contribution to the total cost were laboratory tests, labor, latency time, imaging and consumables. Considering this cost and the current epidemiological data on respiratory infections in France, the absence of valuation for outpatient care represents an annual loss of over 17 million euros for healthcare facilities.

**Conclusion:**

Micro-costing is a key element in valuing healthcare costs. The importance of accurately measuring costs, along with measuring the health outcomes of a defined care pathway, is to enhance the relevance of health economic evaluations and thus ensure efficient care.

## 1. Introduction

In recent years, emergency departments (EDs) have faced major challenges related to overcrowding. In the current ED situation, medico-economic assessments are needed to optimize resource allocation without compromising the quality of care for a growing number of patients (1, 2). Indeed, to make informed healthcare decisions, it is essential to compare both the costs and healthcare outcomes associated with the interventions under consideration (3). Healthcare decision-makers therefore need to identify which processes can be improved, at the right cost, and how this cost relates to health outcomes (4, 5). The accuracy of cost estimates is therefore of paramount importance, as it conditions the evaluation of the effectiveness of strategies. However, estimates of patient care costs are often imprecise and based on overall hospitalization costs, making it difficult to assess the cost of specific emergency care.

It is usual to consider that the choice of costing method involves a balance between accuracy and implementation feasibility (5–7). Costing methods are usually divided into four categories, depending on the monetary valuation of resources (bottom-up versus top-down), and the precision of the measurement (micro-costing versus gross-costing) (6, 8). The micro-costing methods are the most precise costing methods that involve direct, detailed, real-time observation of the resources consumed at each step of each patient’s care (7). It enables the monetization of resources consumption observed at an individual level, thereby facilitating the calculation of a per patient cost of care which will be used to estimate the average cost of the intervention studied. This stands in contrast to the top-down method, which assigns each patient an average cost based on aggregated data (8). While the bottom-up micro-costing method is considered the gold standard for hospital cost estimation due to its precision (3), its practical implementation is often limited due to a significant consumption of labor time and resources (6, 7). The need for detailed data collection at the patient level can be time-consuming and costly, making it less feasible for large-scale studies or healthcare facilities with limited resources. Therefore, despite its accuracy, practical constraints often lead researchers to explore alternative costing methods, such as gross-costing or top-down approaches.

The valuation of healthcare resources consumed is increasingly important in the context of EDs experiencing misuse and overuse. In France, the cost paid to hospitals for outpatient care is based on health insurance reimbursement data, and does not take into account the resources actually consumed during treatment, which certainly underestimates the cost. The extreme situation of the COVID-19 pandemic and the associated hospital saturation, highlighted the importance of setting efficient care pathways within a short time and to rethink care pathways (9, 10). This analysis can be extended to the broader context of respiratory infections ED care. Nevertheless, there is a gap in knowledge when it comes to estimating costs and determining the optimal care pathway for ED outpatients (11). To ensure an accurate cost estimation, a recommended set of steps was proposed (4, 5), guiding the process from selecting a pathology or symptom and defining the patient’s care pathway to calculating the total cost of patient care by estimating the time and cost associated with each step. Based on these recommendations, the main objective of the present study was to accurately estimate the total cost associated with the management of patients presenting with suspected lower respiratory tract infection, a frequent reason for ED visit, in the emergency department of Strasbourg University Hospital (France) by a bottom-up micro-costing method. In this regard, the feasibility of implementing a bottom-up micro-costing technique to calculate the ED care costs has also been assessed. Secondary objectives included identifying the care pathway and cost elements associated with ED care for these patients and assessing the potential financial impact of micro-costing-based ED care cost estimation on healthcare system expenses.

## 2. Methods

### 2.1. Study design, setting, and population

First, the authors conducted a cost analysis using a bottom-up micro-costing method. The primary endpoint was the mean total cost of ED care. A set of recommended steps was used to ensure an accurate cost estimation (4, 5). Thirty patients with a suspected lower respiratory tract infection who visited the ED during business hours were prospectively included between January 1, 2023 and March 31, 2023. To ensure the best representation during this study period, patients were systematically and consecutively included when they arrived at the ED with clinical or historical criteria compatible with a potential lower respiratory tract infection. These criteria included a patient’s history of symptoms such as cough, sputum production, purulent secretions from the airways, shortness of breath, fever when no other infectious cause besides respiratory was initially suspected, chest pain in the absence of other factors suggesting a non-pulmonary origin, a history of asthma or exacerbation of chronic obstructive pulmonary disease, contact with infected individuals, and referral to the ED by a healthcare professional for suspected lower respiratory infection, either based on clinical symptoms or the results of prior complementary examinations conducted before ED admission.

Then, a budget impact analysis was conducted using the findings from this study and data from the French Health Insurance reimbursement tariffs.

### 2.2. Identification of resources

Before conducting the cost analysis, on-site observations were performed to determine the relevant cost elements to be considered. The patient’s clinical pathway steps and resources used during ED care were established in consultation with two expert physicians, validated during on-site observations, and refined iteratively during the cost analysis. The clinical pathway within the Strasbourg ED is presented in Supplementary Figure S1.

### 2.3. Data collection: measurement of resources

The duration of procedures, consumables used and additional investigations were measured through direct observation from patient’s arrival until the medical decision regarding orientation (discharge or hospitalization). The resources used from the medical decision regarding orientation until effective hospitalization or discharge (referred to as latency time) were estimated based on the patient’s electronic medical records.

### 2.4. Valuation of resources

To ensure maximum accuracy, the valuation of resources was based on local unit costs (12). This included staff salaries, the purchase price of drugs, the purchase price of all consumables, and the laboratory’s invoiced price for biological analyses.

The cost of labor was calculated by multiplying the observed time spent on a task (in minutes) by the cost of the human resource(s) involved (in euros/min).

The cost of consumables and laboratory tests was calculated by multiplying the unit cost (in euros) by the number of units consumed.

Since the scanner installed in the ED had already been fully amortized, its utilization had minimal influence on the ED care cost. Therefore, the cost of imaging was estimated for each patient based on the reimbursement tariffs set by the French Health Insurance provided below:

- Chest X-ray: €21.28
- Chest CT scan: €58.77
- Chest CT-scan with contrast: €68.37
- Thorax, abdomen and pelvis CT scan: €84.04
- Thorax, abdomen and pelvis CT scan with contrast: €93.64

The cost of a CT scan includes the procedure fee (€32.00), the technical flat rate (€25.27 for chest CT scan and €50.54 for thorax, abdomen and pelvis CT scan) and the storage cost (€1.50). The use of contrast incurs an additional cost of 9.60€ to which the cost of consumables used for injection must be added.

The cost of equipment was considered negligible therefore not included in the total cost.

All cost elements were integrated into an Excel spreadsheet, allowing for the calculation of the total cost per patient (Supplementary Table S1).

### 2.5. Financial valuation of latency time

The latency time, as referred to in this study, represents the time between the medical decision to hospitalize or discharge a patient, and the patient’s transfer from the ED to the inpatient department or return home.

Based on the patient electronic medical record, the following costs were taken into account for the monetary valuation of resources consumed during the latency time:

- additional laboratory tests conducted,
- consumables and labor required for venipuncture and/or arterial blood gas, as measured during the direct observation process,
- nurse and nursing assistant labor per 8-h shift, using the mean duration of bedside care as observed during the direct observation,
- medical examination per 12-h shift, using the mean duration of clinical examination as measured during the direct observation.

### 2.6. Statistical analysis

At present, there are no specific guidelines regarding the number of patients to include or the minimum level of precision required in micro-costing studies (8). To the best of the authors’ knowledge, there is no micro-costing study in the literature focusing on the costs of ED care for specific clinical pathways. In this study, the authors drew upon existing research conducted using a cost calculation method similar to micro-costing known as Time Driven Activity-Based Costing (TDABC). The decision was made to include 30 patients, aligning with the average number of patients or clinical pathways studied in TDABC research conducted in EDs, which ranges from 8 to 113 procedures (13–16). Traditional frequentist statistical methods cannot estimate the precision of the mean cost calculated through micro-costing, due to absence of existing data in the literature regarding the variance of this cost. The use of Bayesian statistical methods allows for the establishment of a maximum credibility interval range of approximately €500 around the calculated mean when 30 patients are included. This approach relied on estimates of minimum and maximum cost values provided by two expert physicians.

All statistical analyses were conducted using the 4.2.1 version of R statistical software. The quantitative variables were described using the mean, along with the corresponding standard deviation, minimum, and maximum values.

A multiple linear regression was conducted to examine the relationship between the total ED care cost and the various cost elements considered in this study. Assumptions concerning linearity, independence, and distribution were verified. Goodness-of-fit statistics, including BIC and adjusted R-squared, were examined to demonstrate that the selected model was the best fit for the data among the tested models.

A linear regression model was employed to determine the presence of a significant linear association between the total cost and the length of stay in the ED. Assumptions of linear regression were assessed to ensure the validity of the model.

## 3. Results

### 3.1. Care process map

**Figure 1:**
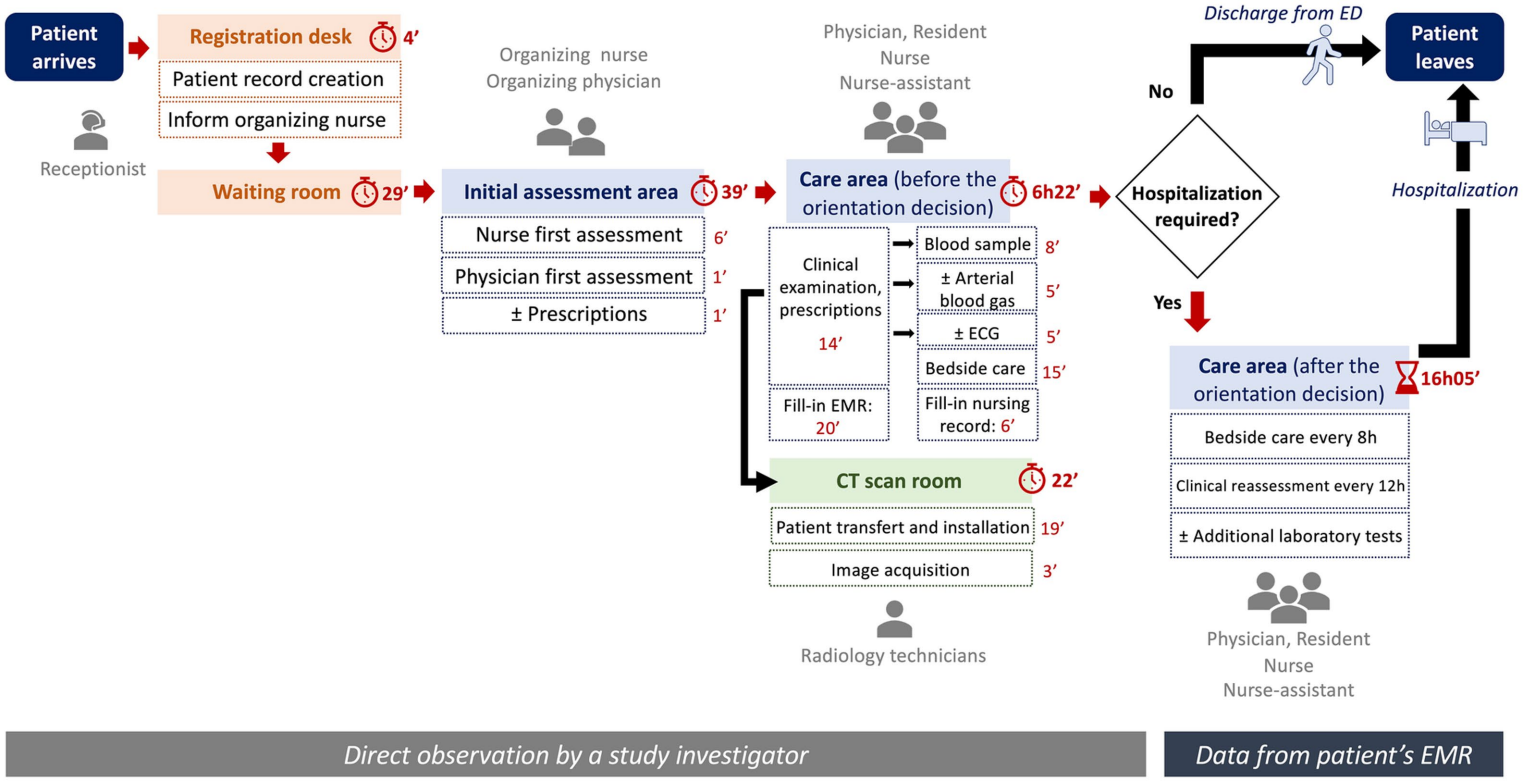
Care process map for a suspected lower respiratory tract infection in the ED. ED, emergency department; min, minutes; h, hours; EMR, electronic medical record; ECG, electrocardiogram.

Direct observation and time measurement were conducted throughout the care pathway for suspected lower respiratory tract infection in the emergency department (ED). The process map, illustrating the various steps and their corresponding durations is represented in Figure 1. The mean duration of ED care prior to the orientation decision was 7 h and 23 min (SD = 3 h and 30 min).

Once the orientation decision was made by the physician, the patient waited an average of more than double that duration (16 h and 5 min, SD = 21 h and 22 min), whether it was for hospitalization or discharge after monitoring and clinical reassessment. The average duration of each step is presented in Table 1.

**Table 1:** Mean duration for each step.

Steps	Mean duration	Minimum duration	Maximum duration	Standard deviation
Waiting room	29 min	1 min	1 h44	35 min
Initial assessment area	39 min	6 min	1 h44	36 min
Nurse first assessment	6 min	2 min	24 min	5 min
Physician first assessment	1 min	1 min	5 min	2 min
Care area	6 h22	1 h16	13 h44	3 h33
Clinical examination	10 min	3 min	25 min	5 min
Bedside care	5 min	1 min	20 min	6 min
Blood sample	8 min	2 min	50 min	9 min
Arterial blood gas	5 min	1 min	25 min	5 min
Monitoring vital signs	5 min	1 min	24 min	5 min
CT scan room	22 min	8 min	1 h34	19 min
Duration of ED care	7 h23	2 h53	18 h19	3 h30

min, minutes; h, hours; CT, computed tomography; ED, emergency department.

### 3.2. Cost elements

The mean total cost of ED care for suspected lower respiratory tract infection, estimated using a bottom-up micro-costing method, is €411.68 euros (SD = 174.49). The cost varied from a minimum of €167.96 to a maximum of €1033.18. The mean cost for each resource category is presented in Table 2.

**Table 2:** Mean cost by type of resource (in euros, €).

Type of resource	Mean cost	Standard deviation
Labor	€90.78	30.82
Receptionist	€1.83	0.64
Paramedical team	€42.87	15.82
Nursing assistant	€3.40	2.56
Nurse	€31.37	13.98
Radiology technician	€8.10	3.93
Medical team	€45.82	20.23
Emergency resident	€24.24	15.42
Emergency physician	€21.58	16.76
Laboratory tests	€155.72	85.57
Blood tests	€87.83	28.32
Microbiological analyses	€67.89	81.78
Consumables	€29.47	21.89
Blood sampling equipment	€4.80	6.03
Microbiological sampling equipment	€18.14	19.14
Wound dressing equipment	€0.11	0.09
Infusion equipment	€1.44	0.96
Oxygenation equipment	€2.21	6.12
Hygiene equipment	€0.62	0.46
Other equipment	€1.31	0.85
Stationery	€0.18	0.03
Imaging	€60.53	25.14
Medication	€4.51	6.02
Latency time cost	€70.67	114.58
Total cost of emergency department care	€411.68	174.49

The most important cost elements were laboratory tests, accounting for 37.8% of the total cost, followed by labor (22.1%), latency time (17.2%), imaging (14.7%) and consumables (7.2%). The cost of medication contributed the least to total cost (1.1%).

To illustrate the relationship between the total cost of ED care (dependent variable, Y) and the cost elements (independent variables, X_i_), a descending stepwise procedure was employed to select the best-fitting model after removing multicollinear variables. The resulting multiple regression model, Y = 180.98 + 0.98×_1_ + 1.09×_2_, where X_1_ and X_2_ represent the cost of laboratory tests and latency time, respectively, exhibited an R-squared value of 0.92. Notably, despite regression coefficients close to 1, there was no evidence of collinearity between the two independent variables (VIF < 1.5). This analysis suggests that 92% of the cost variability observed in this study can be attributed to the costs of laboratory tests and the costs associated with latency time.

### 3.3. Relationship between the cost and the length of stay in the ED

**Figure 2:**
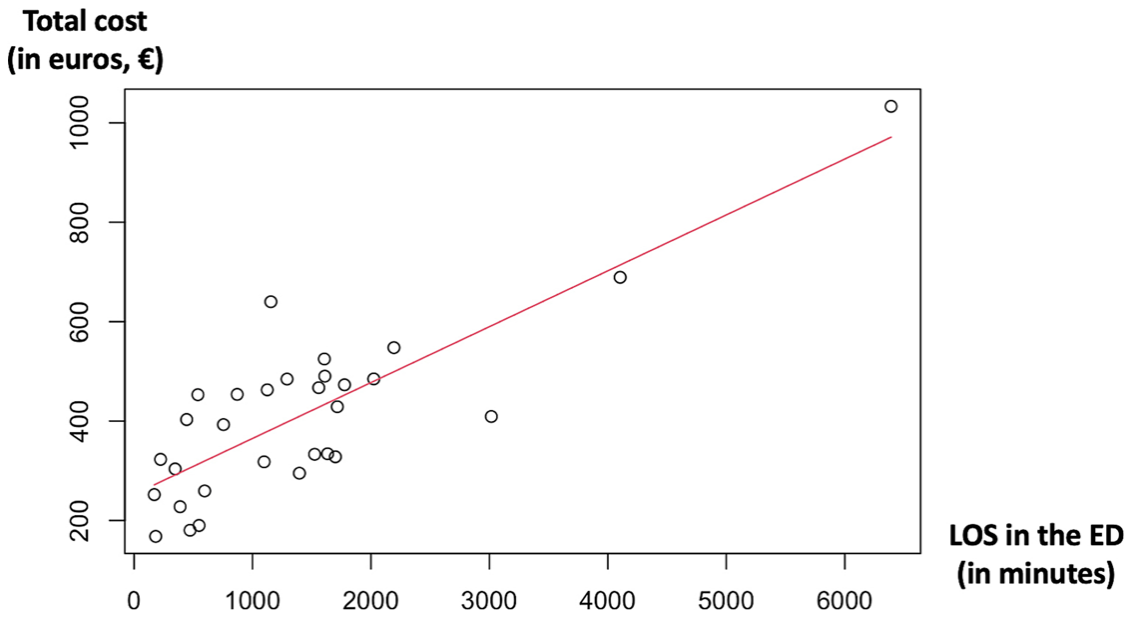
Total cost of ED care versus length of stay in the ED. The line corresponds to the linear regression line. LOS, length of stay; ED, emergency department.

The linear regression model identified a significant linear relationship between the total cost of ED care and the length of stay (LOS) in the ED (β = 0.11, *p* < 0.001), with a coefficient of determination (R-squared) of 68%. These findings suggested that higher LOS in the ED were associated with higher costs. The patient with the longest LOS, specifically 4 days, 10 h, and 31 min, incurred the maximum cost of €1033.18. The distribution of total costs versus LOS is presented in Figure 2.

### 3.4. Budget impact analysis

Each year in France, approximately 500,000 patients are diagnosed with lower respiratory tract infections. Among them, 180,000 will seek care at the ED, and 64% of them require hospitalization. Thus, over 65,000 patients per year are treated as outpatients (17). There is no consensus on the financial assessment of ED care for outpatients, and consequently, no available data on this matter. However, for hospitalized patients, ED care costs are standardized at €150, as part of the Diagnosis-Related Group, calculated using gross-costing methods and used for reimbursement by the French Health Insurance. Subtracting this €150 ED care cost from the one obtained through micro-costing in the present study reveals an additional cost of €261.68. By extrapolating this cost to the 65,000 yearly outpatients, the hospital incurs an annual financial loss exceeding €17,000,000.

## 4. Discussion

This study marks a significant step in healthcare cost analysis, as it is the first comprehensive bottom-up micro-costing study to assess the entirety of care costs associated with patients visiting a French ED for suspected lower respiratory tract infections. In a context where specific data on ED care costs in France are lacking, this research fills a significant gap by providing an initial, highly precise estimate of these costs through the most rigorous calculation method available. Importantly, the successful implementation of this micro-costing approach underscores its feasibility for similar investigations, opening doors to more detailed cost analyses in healthcare settings. However, it’s worth noting that a very small portion of the total cost could not be calculated with the bottom-up micro-costing technique due to equipment depreciation. This situation, which may also arise in other healthcare facilities, leads to considering the development and evaluation of hybrid cost calculation approaches in future studies. While the assumptions made likely have a minimal impact on the overall cost, they should be considered when evaluating the accuracy of the cost estimates.

In addition to its originality, this study reveals how costs elements are distributed throughout the ED care process, offering valuable insights for future resource allocation or, where appropriate, process improvement. It seems that acting on ED latency times and on the prescription of biological tests could represent significant factors for influencing the total cost of ED care. Indeed, the analysis reveals the substantial impact of latency time as the third most important cost element, indicating the strain on hospital bed capacity. A linear relationship was observed between the LOS in the ED and the mean cost of care. To confirm this association, it would be necessary to consider potential confounding factors that influence both the LOS and costs, such as the patient’s condition and the severity of the respiratory infection being treated. These specific data were not collected in this initial exploratory cost-focused study and may be the subject of future research. The budget impact analysis for outpatient costs emphasizes the importance of accurate valuation to ensure appropriate reimbursement for ED services. Given the substantial potential annual loss of over 17 million euros when employing gross-costing methods instead of a bottom-up micro-costing approach for outpatient care, it becomes imperative to acknowledge the broader implications. Inaccurate cost calculation methods that fail to consider actual healthcare facility expenses can compromise healthcare system sustainability, hinder patient access to quality care, and challenge resource allocation strategies. In a healthcare landscape characterized by escalating costs and growing demands, optimizing cost calculations through micro-costing, when proven appropriate, appears crucial to ensure the long-term viability of healthcare systems and equitable access to care for all.

Several studies have demonstrated significant differences between micro-costing and gross-costing methods, with the latter potentially leading to over – or underestimation of costs (18–20). Bottom-up micro-costing provides precise cost estimates by meticulously tracking every resource used in patient care. It offers high accuracy but demands extensive data collection, making it resource-intensive and potentially less feasible for large-scale studies (6, 8). An alternative approach has shown promise in estimating costs of ED care. This method called Time-Driven Activity-Based Costing (TDABC) simplifies cost estimation by assigning standard costs to activities based on time estimates rather than tracking the precise resource consumption associated with each activity, enhancing feasibility and reducing resource consumption (13–16, 21). However, its accuracy may be lower as it relies on approximations. Both methods seem valuable approaches for healthcare cost analysis. For detailed, resource-rich studies, bottom-up micro-costing excels in accuracy. In contrast, TDABC offers a practical compromise when resource constraints or broader-scale analyses come into play. While there is no definitive consensus on the preferred costing method, evidence from the literature suggests that micro-costing methods should be prioritized whenever feasible. Knowing a more precise cost of ED care could encourage decision-makers to distinguish this cost in order to better value the activity of EDs, especially for outpatients.

### 4.1. Limitations

The cost analysis was conducted in a single center, based on a limited number of patients. Since this was an initial feasibility study with an exploratory objective, a sample of 30 patients was deemed adequate, considering the limited available data from precise costing methods such as micro-costing in the literature. Despite the systematic and consecutive inclusion process to ensure the best representation, the study period is limited to regular working hours and the first trimester of the year (January to March). The cost range obtained in this study is only applicable to lower respiratory tract infections occurring during this time of the year and may not correspond to other epidemic peaks or patients admitted to the ED during night shifts. It should be noted that the cost estimation was based on specific local unit costs of Strasbourg University Hospital. It is noteworthy that emergency care protocols for suspected lower respiratory tract infections, along with the complementary diagnostic examinations such as imaging and laboratory tests, typically adhere to standardized guidelines and procedures consistent across healthcare facilities in France. As a result, the cost data collected in this study can offer valuable insights with potential applicability to other hospitals in France, given the standardized nature of care practices in this context. Nevertheless, it is essential to acknowledge that despite the protocolization of certain aspects of emergency care, variations in resource utilization, administrative practices, and local factors may still influence cost profiles. Therefore, while these study findings provide interesting insights, it is important to recognize the potential for variations among hospitals. To apply the findings from this study in different contexts and centers, one should consider seasonality and cost data from the studied hospital (unit prices of consumables, laboratory analysis costs, fully loaded staff salaries, and whether or not imaging equipment is depreciated). The cost data for ED care before the decision on patient orientation can reasonably be replicated by following a similar clinical pathway. These represent standard ED care practices in the context of consultations for suspected lower respiratory tract infections in French EDs. However, the time delay before transfer to the hospitalization department can vary depending on the organization and capacity of the center under study. Calculating this part of the total ED care cost would require specific consideration for each individual center.

Despite the apparent feasibility of employing a bottom-up micro-costing method, it is important to acknowledge that not all costs could be calculated using this technique. This approach aligns with the suggestions of Jacobs et al. and Tan et al., who propose the utilization of multiple costing methods within a single study, thereby disaggregating the total cost into individual cost elements and leveraging the strengths of each method while mitigating their weaknesses (7, 22, 23). Limiting the application of bottom-up micro-costing to cost elements that exert a great impact on the total cost still allows for a reliable estimate of the total cost (6).

It would have been valuable to compare the micro-costing estimate of ED care costs from the present study with previous gross-costing estimates. However, no data in the literature are available for such comparisons with results from similar populations or time periods.

## 5. Conclusion

The study enabled to accurate estimation of the total cost of emergency department (ED) care for patients presenting with suspected lower respiratory tract infection in a French ED, employing a bottom-up micro-costing method.

The study also allowed the identification of the care pathway and cost elements associated with ED care for these patients, and demonstrated the potential financial impact of managing outpatients. If used in policy decisions, these findings could guide cost-effective strategies for managing patients with respiratory infections, improving cost assessment, and optimizing resource allocation. This evidence-based approach could also apply to other common ED visits, contributing to the development of efficient healthcare policies for different conditions.

## Data availability statement

The original contributions presented in the study are included in the article/Supplementary material, further inquiries can be directed to the corresponding author.

## Ethics statement

The studies involving humans were approved by the ethics committee of Strasbourg University Hospital (CE 2021-141). The studies were conducted in accordance with the local legislation and institutional requirements. Written informed consent for participation was not required from the participants or the participants’ legal guardians/next of kin in accordance with the national legislation and institutional requirements.

## Author contributions

VW: Conceptualization, Data curation, Formal analysis, Investigation, Methodology, Project administration, Software, Validation, Visualization, Writing – original draft, Writing – review & editing. É-AS: Methodology, Supervision, Writing – review & editing. PL: Writing – review & editing. ÉB: Investigation, Writing – review & editing. PB: Writing – review & editing. SK: Conceptualization, Methodology, Supervision, Visualization, Writing – review & editing.
