# Supplementary material for: Micro-costing analysis of suspected lower respiratory tract infection care in a French emergency department
Source: Front Public Health. 2023 Oct 4;11:1276373. doi: 10.3389/fpubh.2023.1276373 (PMC10582559; doi:10.3389/fpubh.2023.1276373)
Supplement: Supplementary file 1 [file Data_Sheet_1.DOCX]

Supplementary Material

#
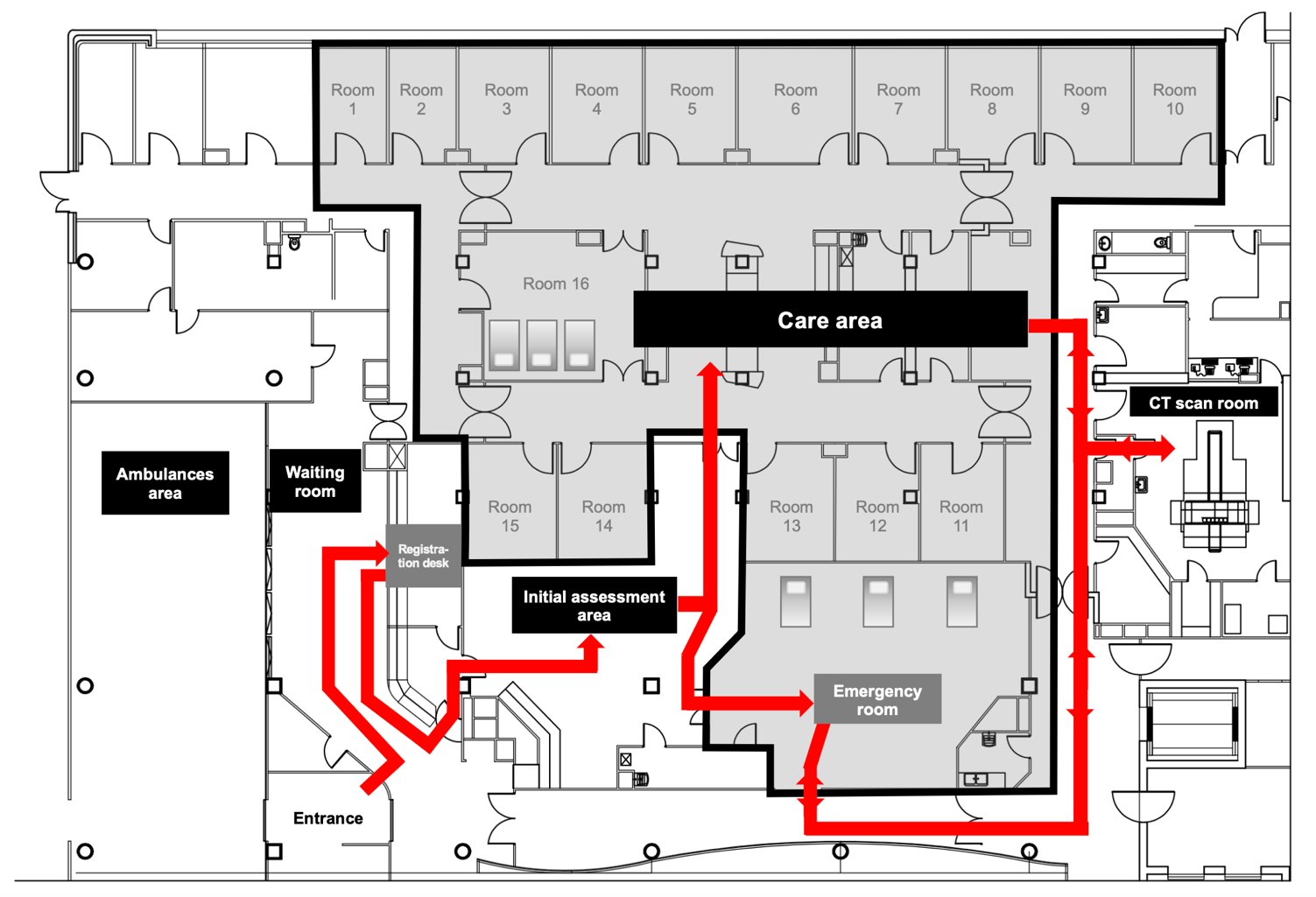
Supplementary Figure 1

**Supplementary Figure 1.** Patient’s clinical pathway in the Emergency Department

CT, computed tomography.

# Supplementary Table 1

**Supplementary Table 1.** Excel micro-costing spreadsheet used for the per-patient cost calculation

aPTT, activated partial thomboplastin time; BNP, B-type natriuretic peptide; CT, computed tomography; ED, emergency department; GGT, gamma glutamyl transferase; GOT, glutamate-oxaloacetate-transaminase; GPT, glutamate-pyruvate-transaminase; h, hour; HCG, human chorionic gonadotropin; INR, international normalized ratio; IU, international unit; IV, intravenous; min, minute; mL, milliliters; NIV, non-invasive ventilation; PCR, polymerase chain reaction; PO, per-os; SAMU, service d’aide médicale urgente (french out-of-hospital acute medical care service); SC, subcutaneous; (T)AP, (thorax), abdomen and pelvis; TSH, thyroid stimulating hormone.
